# Psychosocial status and prenatal care of unintended pregnancies among low-income women

**DOI:** 10.1186/s12884-020-03302-2

**Published:** 2020-10-12

**Authors:** Alanna M. Cruz-Bendezú, Grace V. Lovell, Brianna Roche, Meghan Perkins, Tiffany L. Blake-Lamb, Elsie M. Taveras, Meg Simione

**Affiliations:** 1 Division of General Academic Pediatrics, Department of Pediatrics, Massachusetts General Hospital for Children, 125 Nashua Street, Suite 860, Boston, MA 02114 USA; 2 Department of Obstetrics and Gynecology, MGH, Boston, MA USA; 3 Kraft Center for Community Health Leadership, Partners Healthcare, Boston, MA USA; 4 Department of Nutrition, Harvard T.H. Chan School of Public Health, Boston, MA USA; 5 Department of Pediatrics, Harvard Medical School, Boston, MA USA

**Keywords:** Unintended pregnancy, Maternal mental health, Prenatal care, Low-income women

## Abstract

**Background:**

Nearly half of all pregnancies in the United States are reported as unintended and rates are highest among women of low socioeconomic status. The purpose of this study was to examine the associations between unintended pregnancies and maternal mental health and timing of prenatal care among low-income women.

**Methods:**

In this cross-sectional study, 870 women, whom were participating in the First 1000 Days program in three community health centers in the Boston area, were enrolled at their first prenatal visit from August 2016 – September 2017. We assessed pregnancy intention by self-report using the Pregnancy Risk Assessment Monitoring System. We used self-reported survey information and electronic health record data to assess the following outcomes: current stress, current depression, and timing of initial prenatal visit. We used multivariable logistic regression models to examine associations and adjusted for sociodemographic factors.

**Results:**

Women were a mean (SD) age of 29.3 (6.1), and 39.2% reported that their pregnancy was unintended. 50.6% of women were Hispanic, 28.4% were White, 10.1% were Black, and 10.9% were other races. 78.9% of women reported an annual household income <$50,000. Overall, 26.7% of women reported current stress, 8.2% reported current depression, and 18.3% of women initiated prenatal care after their first trimester. In multivariable analyses, women with unintended pregnancies had higher odds of experiencing current stress (OR: 1.72; 95% CI: 1.22, 2.41), current depression (OR: 1.83; 95% CI: 1.04, 3.20), and initiation of prenatal care post-first trimester (OR: 1.84; 95% CI: 1.23, 2.74).

**Conclusions:**

Unintended pregnancies were associated with current stress and depression, and delayed prenatal care in this sample of low-income women suggesting the importance of identifying high-risk women and tailoring interventions to support women’s needs.

**Trial registration:**

ClinicalTrials.gov (NCT03191591; Retrospectively registered on June 19, 2017).

## Background

Nearly half of all pregnancies in the United States are reported as unintended, defined as unwanted and mistimed pregnancies [1]. Rates of unintended pregnancies are highest among women of low socioeconomic status, as well as among racial and ethnic minorities [2, 3]. Unintended pregnancies are a major public health concern in the United States due to the high incidence and impact on maternal and child health. Unintended pregnancies have been found to be associated with deleterious maternal health behaviors and outcomes, such as inconsistent folic acid consumption, smoking, alcohol intake [4–6]; and poor infant outcomes, such as shortened birth length, low birth weight, and reduced likelihood of breastfeeding [7–10].

In addition, maternal psychosocial outcomes, including stress and depression, and delayed prenatal care have been associated with unintended pregnancies, although few studies examining stress and depression have been conducted during the prenatal period [11]. In a systematic review and meta-analysis, Abajobir and colleagues found a statistically significant association between unintended pregnancies and antenatal and postpartum depression [11]. Less studied than maternal depression, stress has also been found to be associated with unintended pregnancies [12]; although results from other studies reveal mixed findings [11]. Dibaba and colleagues conducted a systematic review and meta-analysis and found that women with unintended pregnancies had greater odds of delaying prenatal care as compared to women with intended pregnancies [13]. Depression, stress, and delayed prenatal care can impact maternal and child health, yet despite the high rates of unintended pregnancies among women of low socioeconomic status, few studies or reviews have further examined these outcomes in a low-income population [14].

A more detailed understanding of these outcomes in a low-income population would allow for improved identification of women at high risk for poor outcomes and the development of tailored approaches to prenatal care. The purpose of this cross-sectional study was to examine maternal mental health and prenatal care as correlates of unintended pregnancies among low-income women enrolled in the First 1000 Days program in the Greater Boston area. We hypothesized that women who reported having an unintended pregnancy would be more likely to report adverse mental health status and would be more likely to initiate prenatal care after their first trimester compared to women with intended pregnancies.

## Methods

The First 1000 Days program is a systems-level initiative that engages stakeholders across clinical and public health sectors to reduce the prevalence of obesity and obesity risk factors among low-income mother-infant pairs by addressing the various levels of individual, family, and socio-contextual factors that impact obesity prevention [15]. We implemented the First 1000 Days program at three community health centers in the Greater Boston, MA area, Massachusetts General Hospital (MGH) Revere HealthCare Center, MGH Chelsea HealthCare Center and DotHouse Health, whose populations are primarily composed of low-income, racial/ethnic minority, primarily publicly insured, and foreign-born patients. Pregnant women enrolled in the First 1000 Days program if they received their obstetrics care at one of the three health centers and completed the program’s intake survey on an iPad or paper at their initial nurse prenatal visit, which was available in English, Vietnamese, Spanish and Arabic. The initial prenatal intake survey was offered to all women who initiated prenatal care at the three health centers between August 2016 and September 2017. At the initial prenatal intake visits, women had the option to complete the intake screener or decline participation. A small proportion of women who were not offered the intake screener by the clinical staff at the time of their appointment or were not able to complete the intake survey by paper were later contacted by phone and given the option to complete the intake screener and enroll in the program. For women who completed multiple intake surveys during the study period (e.g. women with an early pregnancy loss and a subsequent pregnancy), only the first intake survey was used. The study protocol was approved by the Partners Healthcare institutional review board and registered at ClinicalTrials.gov (NCT03191591).

### Pregnancy intention

The main exposure for this study, which was a secondary analysis, was pregnancy intention, assessed through the initial prenatal intake survey. We asked women, “Thinking back to when you learned you were pregnant, how did you feel about being pregnant?” Women had a choice of the following responses: “I wanted to be pregnant sooner”, “I wanted to be pregnant later”, “I wanted to be pregnant now”, or “I didn’t want to be pregnant now or at any time in the future”. This survey question is from the Center for Disease Control’s Pregnancy Risk Assessment Monitoring System (PRAMS) [16]. We classified responses into two categories: intended pregnancy for women responding, “I wanted to be pregnant sooner” or “I wanted to be pregnant now”, and unintended pregnancy, which included mistimed and unwanted pregnancies, for women responding, “I wanted to be pregnant later” or “I didn’t want to be pregnant now or at any time in the future.” This classification has been widely used across similar studies and analyses [8, 10, 17].

### Current stress

To measure current stress, we asked women “How much stress do you feel in your life?” which was adapted from the Growing Up Today Study [18]. We defined current stress as any positive responses including “I feel stress fairly often”, “I sometimes feel a lot of stress” or “I feel a lot of stress most of the time”.

### Current depression

Current depression was measured using the Patient Health Questionnaire-2 (PHQ-2) [19] or the Edinburgh Postnatal Depression Scale (EPDS) [20] depending on the health center. Mothers who completed the initial prenatal intake survey at DotHouse Health answered the PHQ-2 questions “Over the past two weeks, how often have you been bothered by any of the following problems?” and “Over the past two weeks, how often have you been feeling down, depressed, or hopeless?” We categorized a score ≥ 3 on the PHQ-2 as current depression [19]. For those women who completed the prenatal intake survey at the MGH Chelsea and MGH Revere HealthCare Centers, we obtained an EPDS score from their electronic health record (EHR) to assess current depression. We defined an EPDS score ≥ 12 and/or a reported answer other than “never” when asked if “The thought of harming myself has occurred to me” within the past 7 days, as current depression [20].

### Gestational age at initial prenatal visit

We established when participants began seeking prenatal care by obtaining gestational age at their initial prenatal visit from the EHR. The American Academy of Pediatrics and the American College of Obstetricians and Gynecologists Guidelines for Perinatal Care recommend that women begin their prenatal care within the first trimester [21, 22], and we defined the start of care within the first trimester of pregnancy as early initiation and the start of care any time after the first trimester as late initiation (after 13 weeks).

### Confounding factors

We selected covariates based on the literature and collected information regarding these factors from the prenatal intake survey and the EHR. From the intake survey, we collected maternal race/ethnicity (White; Hispanic or Latino; Black; Asian or Other) [1, 2, 17, 23], annual household income [1–3], marital status, primary care visit within the past 12 months, and country of origin [2, 7, 24]. From the EHR, we collected age in years [1, 2, 10], gravidity [2, 10, 24], and health insurance coverage at the time of survey completion [2, 25].

### Data analysis

Participant characteristics were described overall and according to pregnancy intention status. We used χ^2^ tests to compare categorical variables and t-tests to compare continuous variables by pregnancy intention status. Multivariable logistic regression was used to determine the odds ratios and 95% confidence intervals for each of the three outcome variables: experiencing current stress, screening positive for current depression, and initiating prenatal care after the first trimester. Results from all models were adjusted for confounders chosen a priori after a review of the literature. For each outcome, the following series of models were created. Model 1 adjusted for race/ethnicity and maternal age. Model 2 adjusted for all covariates in model 1 plus household income, marital status, and country of birth. Model 3 adjusted for all covariates in model 2 plus gravidity and insurance status. For analyses, we used Model 3. A 2-sided alpha level of 0.05 was used to test for statistical significance in all analyses. All analyses were performed using R version 3.4.4 [26].

### Missing data

Among the women who completed the maternal intake screener at their initial prenatal visit, 73.4% had no missing data for pregnancy intention, covariates, and outcomes. Overall, 3.2% of the dataset was missing in fully-adjusted models, with missing percentage for individual variables ranging from less than 2% for maternal age to 12% for depression screening. We excluded women from the analyses who did not answer the pregnancy intention question (14 participants). In order to retain all available observations, minimize bias due to missing information, and improve efficiency in parameter estimation, we used multivariate imputation by chained equations implemented with the mice package [27, 28]. We first visualized the missingness patterns for each variable and decided that the missing at random assumption was reasonable. Next, we specified the imputation model—logistic regression for dichotomous variables, multinomial logistic regression for categorical variables, and predictive mean matching for continuous variables. We included the following variables in the order listed in the imputation model: health care center of prenatal care initialization, language, calendar year and month, household size, public benefits enrollment, pre-pregnancy body mass index, housing and food insecurity status, age, gravidity, gestational age at initial prenatal visit, race, income, insurance status, marital status, country of origin, pregnancy intention, current stress, current depression, and primary care engagement in the twelve months before initializing prenatal care. Twenty iterations were used to ensure convergence and 30 multiply imputed data sets were created. The results for the multivariable logistic regressions were obtained by combining the separate estimates and standard errors from each of the 30 imputed datasets.

## Results

Eight hundred seventy women were included in the final statistical analyses. Women were a mean (SD) age of 29.3 (6.1) years, and 39.2% reported that their pregnancy was unintended. 50.6% of women were Hispanic, 28.4% were White, 10.1% were Black, and 10.9% were other races. 78.9% of women reported an annual household income <$50,000. Overall, in this study, 26.7% of women reported current stress and 8.2% reported current depression. 18.3% of women initiated care after their first trimester. For the women reporting unintended pregnancy, 57.4% identified as Hispanic or Latino, had a mean (SD) age of 27.7 (6.5) years, and over 80% made <$50,000 a year. 32.2% of women reporting unintended pregnancy had a marital status of “other”, 78.8% had public insurance, and 38.5% did not have a primary care visit within the last 12 months. Women reporting unintended pregnancy were younger, not married, more likely to have public insurance, and less likely to have attended a primacry care visit as compared to women reporting intended pregnancy. Furthermore, 34.4% reported current stress, 12% reported current depressive symptoms, and 24.7% had initiated care after the first trimester. Whereas, women whom reported an intended pregnancy, 21.7% had current stress, 5.81% had current depression, and 14.2% had initiated care after the first trimester. Table 1 presents the maternal characteristics and bivariate associations by the overall study sample and pregnancy intention.

**Table 1:** Maternal characteristics at initial prenatal care visit and outcomes by pregnancy intention

	Overall	Intended Pregnancy	Unintended Pregnancy	***p***-value
	***N*** = 870	***N*** = 529	***N*** = 341	
***Participant Characteristics, Mean (SD) or n (%)***				
Average Age, years	29.3 (6.1)	30.3 (5.5)	27.7 (6.5)	**<.001**
Race/Ethnicity				.02
White	235 (28.4)	156 (30.7)	79 (24.7)	
Hispanic or Latino	419 (50.6)	236 (46.5)	183 (57.2)	
Black	84 (10.1)	59 (11.6)	25 (7.8)	
Other	90 (10.9)	57 (11.2)	33 (10.3)	
Annual Household Income				**<.001**
< $10 k/year	127 (16.5)	61 (12.9)	66 (22.0)	
$10 k-$20 k/year	181 (23.4)	113 (23.9)	68 (22.7)	
$20 k-$50 k/year	301 (39.0)	175 (37.1)	126 (42.0)	
> $50 k/year	163 (21.1)	123 (26.1)	40 (13.3)	
Marital Status				**<.001**
Married/living together	693 (80.6)	464 (88.9)	229 (67.8)	
Other	167 (19.4)	58 (11.1)	109 (32.2)	
Place of Birth				.06
United States	307 (36.1)	174 (33.5)	133 (40.2)	
Other	544 (63.9)	346 (66.5)	198 (59.8)	
Gravida	2.51 (1.5)	2.47 (1.4)	2.57 (1.6)	.33
Health Insurance Status				**.01**
Commercial	239 (27.5)	167 (31.6)	72 (21.2)	
Free care	21 (2.4)	12 (2.3)	9 (2.7)	
Medicaid/Government	512 (59.0)	296 (56.0)	216 (63.7)	
Self-pay	96 (11.1)	54 (10.2)	42 (12.4)	
Primary care visit within the last 12 months				**.01**
Yes	599 (69.2)	380 (72.5)	219 (64.2)	
No	266 (30.8)	144 (27.5)	122 (35.8)	
***Measured Outcomes, n (%)***				
Current Stress				**<.001**
Experiencing stress	229 (26.7)	113 (21.7)	116 (34.4)	
Little or no Stress	629 (73.3)	408 (78.3)	221 (65.6)	
Current Depression				**.004**
Experiencing depression	63 (8.2)	27 (5.81)	36 (12.0)	
No depression	703 (91.8)	438 (94.2)	265 (88.0)	
Gestational Age at Initial Prenatal Visit				**<.001**
Initiated care after first trimester	154 (18.3)	73 (14.2)	81 (24.7)	
Initiated care within the first trimester	689 (81.7)	442 (85.8)	247 (75.3)	

In unadjusted models (Table 2), we found that women with unintended pregnancies had higher odds of experiencing current stress (OR: 1.91; 95% CI: 1.40, 2.59), current depressive symptoms (OR: 2.09; 95% CI: 1.24, 3.51), and initiating prenatal care after the first trimester (OR: 1.93; 95% CI: 1.36, 2.74) compared to women with intended pregnancies.

**Table 2:** Associations of Maternal Mental Health and Prenatal Care with Unintended Pregnancies, Compared to Intended Pregnancies, in Unadjusted and Multivariable Adjusted Models

	Unadjusted Models	Fully Adjusted Multivariable Models^a^
	OR (95% CI)	OR (95% CI)
Experiencing current stress	1.91 (1.40, 2.59)	1.72 (1.22, 2.41)
Experiencing current depression	2.09 (1.24, 3.51)	1.83 (1.04, 3.20)
Initiated prenatal care after 1st Trimester	1.93 (1.36, 2.74)	1.84 (1.23, 2.74)

Reference group = Intended pregnancy. *OR* Odds ratio, *CI* Confidence interval

^a^Model adjusted for race/ethnicity, maternal age, household income, marital status, country of birth, gravidity, and insurance status.

In the fully adjusted multivariable models adjusting for race/ethnicity, maternal age, household income, marital status, country of birth, gravidity, and insurance status, the associations persisted albeit mildly attenuated between the main exposure, pregnancy intention and the psychosocial outcomes (Table 2). As compared to women with intended pregnancies, women with unintended pregnancies had higher odds of experiencing current stress (OR: 1.72; 95% CI: 1.22, 2.41) and current depressive symptoms (OR: 1.83; 95% CI: 1.04, 3.20); and they had higher odds of initiating prenatal care after the first trimester (OR: 1.84; 95% CI: 1.23, 2.74).

## Discussion

In this study of 870 low-income women seeking prenatal care, we found that close to 40% reported an unintended pregnancy. We found that women with unintended pregnancies had higher odds of experiencing current stress and depression; and initiating prenatal care after the first trimester as compared to women with intended pregnancies. The findings were robust when adjusted for sociodemographic factors that might confound the associations between pregnancy intention and our outcomes. Understanding these relationships can aid in improved methods for the identification of women whom may be at risk for stress, depression, and delayed prenatal care. By studying women of low socioeconomic status, who are known to have high rates of unintended pregnancies [2, 3], we can tailor interventions during pregnancy to address mental health and timely prenatal care, as well as other known barriers that will help improve maternal and child health outcomes.

Our study found an association between unintended pregnancies and current stress and depression; similarly, other studies have found an association between pregnancy intention and prenatal depression and stress. These studies assessed depression and stress around the time of delivery, and when compared to our study participants, participants were predominately high-income and less racially and ethnically diverse [12, 29]. A study by Maxson & Miranda [30], which also found an association between pregnancy intentions and depression and stress, sampled low-income women and completed assessments between 18 and 28 weeks gestation, which was slightly later than the collection time period for our study. The strength of association in our study was found to be stronger than in the Maxson & Miranda study, which may be explained by the differing collection time periods during pregnancy or the collection dates. Our data were collected between 2017 and 2018, whereas their data were collected between 2005 and 2010, which may indicate stress and depression is becoming an increasing concern for women with unintended pregnancies.

We also found an association between unintended pregnancies and timing of initiating prenatal care. Our finding replicates what has been previously found in the literature [8, 23], and extends the findings to women whom are low-income and racial and ethnic minorities, which few studies have examined. Orr and colleagues found that women whom were black and living in an urban area with unintended pregnancies were more likely to initiate prenatal care in their third trimester [14]. In our study population, which was compromised of women whom approximately 50% were Hispanic and approximately 79% earned less than $50,000 per year, we found women with unintended pregnancies also delayed care until after the first trimester. For some women, relationship status may have also contributed to household income, as 32% of women who reported unintended pregnancy were not married.

In this cross-sectional, secondary analysis, we did not assess women’s stress, depression, or other life circumstances prior to conception or during the postnatal period or their attitudes towards the pregnancy. Studies suggest that the relationship between pregnancy intention and stress and depression is bidirectional [31, 32]. Some women might have been experiencing stress and depression prior to conceiving which may then have exacerbated symptoms during pregnancy. Women may experience differing emotions when they recognize they are pregnancy; the evidence suggests that unintended pregnancies that women view as more “acceptable” have stronger correlations to positive maternal and child health outcomes [33, 34]. For women living in low-income communities, structural and institutional factors play a critical role in accessing healthcare during women’s reproductive years impacting pregnancy intentions [35, 36]. In our study, 30.8% of women had not received primary care within a year before initiating prenatal care. The lack of access and engagement may perpetuate feelings of stress and depression and influence when prenatal care is initiated [34]. Massachusetts has had universal health coverage since 2006 and a majority of women in our study were insured, thereby suggesting other factors besides lack of insurance coverage impacted women’s primary care engagement and timing of prenatal care.

Our study presents with several limitations. As previously discussed, our study was cross-sectional and we were not able to draw conclusions about the causality of the associations and what variables (i.e., stress or depression) may have preceded the pregnancy. We used questionnaires that were administered during pregnancy and have been widely used for measuring pregnancy intentions and maternal psychosocial status. We did not, though, measure women’s attitudes towards their unexpected pregnancies. In addition, our study population does not include women with unintended pregnancies who may have either miscarried, terminated their pregnancy before initiating prenatal care, or never sought prenatal care. We acknowledge the importance of these topics and the ongoing discussions in the field on how to best measure pregnancy intention and reproductive autonomy [34, 37], but the data examined in this study still provides important information about pregnancy intentions in specific populations.

## Conclusions

We found that approximately 40% of women in this study whom were predominately low-income reported an unintended pregnancy. Women with unintended pregnancies had higher odds of experiencing current stress and current depression and were also found to delay their initial prenatal care until after their first trimester. These findings demonstrate the importance of identifying women whom may be at high risk of adverse consequences and ensuring interventions during the prenatal time period appropriately support women in addressing stress, depression, and accessing timely prenatal care.

## Data Availability

The datasets used during the current study are available from the corresponding author on reasonable request.

## Abbreviations

MGH: Massachusetts General Hospital
PRAMS: Pregnancy Risk Assessment Monitoring System
PHQ-2: Patient Health Questionnaire-2
EPDS: Edinburgh Postnatal Depression Scale
EHR: Electronic health record
OR: Odds ratio
CI: Confidence Interval
